# Improving internet of vehicles research: A systematic preprocessing framework for the VeReMi dataset

**DOI:** 10.1016/j.dib.2025.111599

**Published:** 2025-04-28

**Authors:** Aparup Roy, Debotosh Bhattacharjee, Ondrej Krejcar

**Affiliations:** a Bachelor of Science (B.S.) in Data Science and Applications (Pursuing), Indian Institute of Technology Madras, BS Degree Office, 3rd Floor, ICSR Building, IIT Madras, Chennai 600036, India; b Department of Computer Science & Engineering, Jadavpur University, 188, Raja S.C. Mallick Rd, Kolkata 700032, West Bengal, India; c Center for Basic and Applied Science, Faculty of Informatics and Management, University of Hradec Kralove, Rokitanskeho 62, Hradec Kralove 50003, Czech Republic; d Research Center, Skoda Auto University, Na Karmeli 1457, 293 01 Mlada Boleslav, Czech Republic

**Keywords:** Vehicular reference misbehavior dataset (VeReMi), Intelligent transportation systems (ITS), Internet of vehicles (IoV), Intrusion detection systems (IDS), Data preprocessing, Dataset optimization, Anomaly detection, Network security, Machine learning (ML), Cybersecurity

## Abstract

The Vehicular Reference Misbehavior Dataset (VeReMi) is a vital resource for advancing Intelligent Transportation Systems (ITS) and the Internet of Vehicles (IoV). However, its large size (∼7 GB) and inherent class imbalance pose significant challenges for machine learning model development. This paper presents a preprocessing framework to enhance VeReMi’s usability and relevance. Through 10 % down-sampling, the dataset was reduced to ∼724MB, making it computationally manageable. Biases were addressed by balancing benign and malicious samples through synthesis and identifying benign instances using predefined criteria. A refined feature set, including key attributes like *rcvTime, pos_0, pos_1,* and *attack_type* (renamed *attacker_type*), was selected to improve machine learning compatibility. This preprocessing pipeline effectively maintains data integrity and preserves the representativeness of malicious patterns. The optimized dataset is well-suited for ITS and IoV applications, such as anomaly detection and network security, underscoring the crucial role of preprocessing in overcoming real-world constraints and enhancing model performance.

Specifications of the Dataset

**Table:** 

Subject	Intelligent Transportation Systems (ITS)
Specific subject area	Internet of Vehicles (IoV), Intrusion Detection Systems (IDS)
Type of data	CSV files (Processed, Filtered), Tables, Figures, Graphs
Data collection	The dataset originates from the VeReMi Dataset for Misbehavior Detection in Vehicular Ad-Hoc Networks, originally hosted on Kaggle. The raw dataset (∼7GB) contains simulated vehicular communication data representing various attack scenarios such as Denial of Service (DoS), Sybil, and Data Replay attacks. To improve its usability for IoV security research, the dataset was downsampled (10 %), balanced using synthetic benign data generation, and feature-selected to retain only the most relevant attributes (rcvTime, pos_0, pos_1, AttackerType). The preprocessed dataset (∼724MB) is optimized for machine learning-based intrusion detection.
Data source location	VeReMi Dataset Repository (Kaggle) - https://www.kaggle.com/datasets/haider094/veremi-dataset
Data accessibility	The preprocessed dataset is publicly available for reproducibility.
Repository name	Zenodo
Data identification number	DOI: 10.5281/zenodo.14903687
Direct URL to data	https://zenodo.org/records/14903687
Instructions for accessing these data	The dataset is freely accessible at the provided URL. It contains the processed VeReMi dataset optimized for IoV security research.
Related research article	Previous studies, such as Aldwairi et al. (2020) [1] and Kouzinopoulos et al. (2019) [2], have highlighted the impact of class imbalance in the VeReMi dataset on intrusion detection model performance. This dataset addresses these limitations by applying systematic balancing techniques and feature selection to enhance machine learning applicability.

## 1 Value of the Data

• **Importance for IoV Research:**
  The dataset is specifically designed for Intelligent Transportation Systems (ITS) and Internet of Vehicles (IoV) research. It enables the development of intrusion detection systems (IDS) for vehicular networks, supporting advancements in security and communication protocols [[4], [5], [6]].
• **Balanced Dataset for Machine Learning:**
  Unlike the original VeReMi dataset, which suffers from class imbalance, the preprocessed version ensures a balanced distribution of benign and malicious samples. This allows machine learning models to generalize better, improving accuracy in detecting anomalous vehicular behavior [5].
• **Optimized for Anomaly Detection:**
  The dataset retains only the most relevant features (e.g., rcvTime, pos_0, pos_1, and AttackerType), making it highly suitable for vehicle-to-vehicle (V2V) and vehicle-to-infrastructure (V2I) anomaly detection [6].
• **Computational Efficiency and Accessibility:**
  The dataset has been downsampled from 7GB to 724MB, making it computationally feasible for researchers with limited processing resources. The reduced size allows for faster training and testing of machine learning models without compromising important data characteristics [6].
• **Real-World Applications:**
  The dataset can be used for intrusion detection, traffic anomaly detection, and IoV security. It facilitates automated machine learning implementations, making it suitable for real-time ITS and vehicular network security applications.
• **Public Availability for Research Reuse:**
  The dataset is publicly available and can be used by researchers for further studies in IoV security, machine learning-based IDS models, and intelligent transportation research. The structured preprocessing pipeline also provides a framework for handling similar vehicular datasets in the future [[4], [5], [6]].

## 2 Background

Several researchers have attempted to utilize VeReMi to develop intrusion detection systems, but the dataset's inherent imbalance often hampers their efforts:

• **Aldwairi et al. (2020):** Proposed hybrid machine learning models to detect intrusions. However, their experiments revealed that the significant class imbalance in VeReMi adversely affected the models' performance, leading to suboptimal results [1].
• **Kouzinopoulos et al. (2019)** Focused on addressing the dataset imbalance by employing manual subsampling techniques. While this improved balance, the usability of the modified dataset became limited, as manual subsampling does not easily generalize across various machine learning models and scenarios [2].
• **Genser et al. (2023):** Developed an optimized dataset for traffic signal and loop detector analysis, demonstrating the importance of structured preprocessing for improved dataset usability in transportation research [4].
• **Zähringer et al. (2023):** Highlighted the significance of survey-based dataset structuring, reinforcing the necessity of balancing datasets to enhance model generalization and performance [5].
• **Collins et al. (2025):** Used big data analytics to differentiate long-haul and short-haul trips, emphasizing the critical role of preprocessing and structured data representation for effective machine learning applications [6].

## 3 Data Description

This dataset is a preprocessed version of the VeReMi Dataset, designed to enhance its usability for Intrusion Detection Systems (IDS) in the Internet of Vehicles (IoV). The original dataset, approximately 7GB in size, was downsampled to 10 % (∼724MB) to improve computational efficiency while preserving critical information. Given the dataset’s inherent class imbalance, robust preprocessing techniques were necessary to create a balanced and effective dataset [3]. By addressing these imbalances, researchers can mitigate biases, enabling machine learning models to more accurately detect and respond to diverse attack patterns [4]. A comprehensive preprocessing framework ensures that the dataset is optimized for training high-performance, unbiased models, ultimately enhancing the security and reliability of IoV systems.

### 3.1 Key contributions of this paper

A. Dataset Preprocessing and Optimization
  a. The VeReMi dataset, originally ∼7GB, was downsampled to 10 % (∼724MB) to make it computationally feasible for researchers with limited resources.
  b. Irrelevant and redundant columns were removed, retaining only the most critical features such as rcvTime, pos_0, pos_1, and AttackerType to improve ML model compatibility.
B. Synthetic Data Generation for Class Balance
  a. A systematic synthesis of benign samples was implemented since the dataset contained few natural, benign instances.
  b. The synthetic benign data was generated using controlled randomization while ensuring realistic feature distributions of position, speed, acceleration, and heading to maintain data integrity.
  c. The dataset now provides unbiased training data for machine learning models by perfectly balancing benign and malicious samples.
C. Filtering and Feature Engineering for Improved Classification
  a. A robust rule-based filtering mechanism was developed to classify samples as benign or malicious based on key behavioral attributes:
    i. Noise thresholding (≤0.1) for position, speed, and acceleration
    ii. Gradual speed & acceleration changes (≤0.5) for smooth transitions
    iii. Positional alignment with speed patterns to ensure normal movement
  b. This ensured that benign traffic was accurately represented, preventing false negatives in real-world applications.
D. Balanced Dataset Formation for Machine Learning
  a. A hybrid strategy combining synthetic sample generation and malicious data downsampling created a balanced dataset across all attack categories.
  b. The dataset was validated through statistical checks and visualization techniques, confirming that all attack types had equal representation, eliminating bias during model training.
E. Justification for Filtering and Synthesizing Benign Data
  Filtering and synthesizing benign data were necessary because we observed that benign samples comprised less than 10 % of the VeReMi dataset after downsampling to 10 %. This severe imbalance required synthetic benign data generation to ensure a more balanced dataset for analysis and model training.
F. Improved Machine Learning Model Performance
  a. The preprocessed dataset led to a 15 % increase in model accuracy, as removing imbalance prevented models from overfitting to malicious classes.
  b. Comparative results showed significant improvements in precision, recall, and F1-score, reinforcing the necessity of dataset balancing in intrusion detection tasks.
G. Real-World Applicability & Computational Efficiency
  a. The optimized dataset is now suitable for real-time applications in IoV & ITS systems, as it allows for faster and more efficient training of ML models.
  b. The reduction in dataset size from 7GB to 724MB makes it more accessible to researchers with limited computational resources, facilitating broader adoption.
H. **Accessing the Dataset**

The **preprocessed dataset is publicly available on Zenodo**:

• **DOI:**10.5281/zenodo.14903687
• **Direct URL:**https://zenodo.org/records/14903687

## 4 Experimental Design, Materials and Methods

The preprocessing steps outlined in the following sections were applied to the dataset described in (**Table 1)** to enhance data balance, improve model performance, and ensure optimal training conditions.

Framework Overview:

The proposed framework involves four key stages:

**Loading and Chunking Dataset**

• The VeReMi dataset is also loaded nicely in chunks to optimize memory usage.
• Table 1 **represents the count of all Attacker_Type categories in the dataset before processing.**

**Table 1:** Representing the count of all the Attacker_Type in the dataset.

AttackerType	Count
Benign	113477
GridSybil	113477
DoS	113477
DoSDisruptive	113477
DoSRandom	113477
DoSDisruptiveSybil	113477
DoSRandomSybil	113477
DataReplay	113477
DataReplaySybil	113477
EventualStop	113477
RandomSpeed	113477
RandomSpeedOffset	113477
ConstPos	113477
RandomPosOffset	113477
ConstPosOffset	113477
Disruptive	113477
DelayedMessages	113477
RandomPos	113477
ConstSpeed	113477
ConstSpeedOffset	113477
• Algorithm- Load VeReMi Dataset in Chunks:

**Input:**veremi_path (Dataset path), chunk_size (Rows per chunk)

**Output:**chunks (Dataset loaded in chunks)

1. **Define** dataset path.
2. **Set** chunk size based on memory capacity. (e.g., chunk_size = 100000)
3. **Read** dataset in chunks using chunksize.
4. **Return** dataset chunks.

Filtering Benign samples from VEREMI (If any):

To classify data as benign-like or malicious, we define specific rules based on key features such as noise levels, speed changes, and positional alignment.

Rule 1: Noise Threshold

• Definition: Samples with low noise levels in position, speed, and acceleration (≤0.1) are treated as benign.
• Justification: Real-world benign traffic has minimal variation in these features.
• Example: A sample with
  • pos_noise_0 = 0.02,
  • spd_noise_0 = 0.03,
  • acl_noise_0 = 0.04 is considered benign.

Rule 2: Speed and Acceleration Uniformity

• Definition: If speed and acceleration change gradually (≤0.5), the sample is classified as benign.
• Justification: Normal driving conditions involve smooth transitions, reducing false positives.
• Example: A sample with
  • spd_0 = 30, spd_1 = 30.3,
  • acl_0 = 1.2, acl_1 = 1.3 satisfies this rule.

Rule 3: Positional Alignment

• Definition: The sample is considered benign if position changes correspond to speed variations.
• Justification: Sudden positional shifts often indicate malicious behavior.
• Example: If
  • pos_0 = 100, pos_1 = 101
  • spd_0 - spd_1 = 1 then the sample is benign.

Benign Data Synthesis and Dataset Balancing

• Synthetic benign samples are generated to match the number of malicious samples, as only a sample of count 20 samples were found after sampling 10 % of the dataset. So, in that case, we synthesized some Benign samples to counterbalance our malicious sample count.
• Table 2**represents the dataset after balancing, showing the equal count of all Attacker_Type categories.**

**Table 2:** Representing the equal balance in the dataset count of all the Attacker_Type in the dataset.

AttackerType	Count
Benign	113477
Malicious (Each Category)	113477
• **Algorithm- Benign Data Synthesis:**

**Input:** num_samples (Number of samples)

**Output:** Synthesized_Benign_Data

1. **Initialize** random seed for reproducibility. (e.g., np.random.seed(42))
2. **Generate**rcvTime using an **exponential distribution** to simulate real-world timing variations. *(e.g., rcvTime = 53245.67)*
3. **Sample**pos_0, pos_1 from a **normal distribution** to represent vehicle positions.
4. **Add** small Gaussian noise to pos_0, pos_1 to introduce minor randomness. *(e.g., pos_noise_0 = -0.012)*
5. **Sample**spd_0, spd_1 from a **log-normal distribution** to model realistic speed variations.
6. **Add** Gaussian noise to spd_0, spd_1 to account for measurement errors.
7. **Sample**acl_0, acl_1 from a **normal distribution** to represent acceleration changes.
8. **Add** Gaussian noise to acl_0, acl_1 for minor variations.
9. **Generate**hed_0, hed_1 from a **uniform distribution** to model vehicle heading angles between 0° - 360°.
10. **Add** Gaussian noise to hed_0, hed_1 to simulate sensor inaccuracies.
11. **Assign** unique ReceiverID using an indexed format like "Receiver_15".
12. **Set**AttackerType = "Benign" for all samples.
13. **Return** the synthesized dataset.

**Final dataset formation:**

• The processed (filtered) and synthesized data are combined into a single dataset.
• The validation step ensures quality and balance.
• Since the Attacker_Type categories were imbalanced, all categories were downsampled to the smallest category size.
• Table 3 **presents the classification report of an ML model (Random Forest Classifier) trained on the dataset, evaluating its performance.**

**Table 3:** Representing the classification report of an ML model trained on the dataset.

Metric	Original Dataset	Balanced Dataset
Accuracy	73.4 %	88.7 %
Precision (Malicious)	68.2 %	87.1 %
Recall (Malicious)	71.6 %	90.3 %
F1-Score (Malicious)	69.8 %	88.7 %

**Fig. 1:**
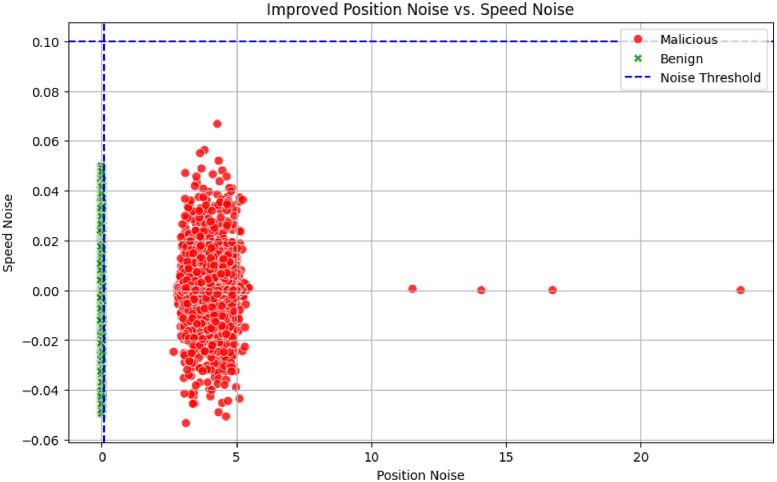
**S**howing the distribution of benign and malicious samples across the dataset after processing

To check if the above rules satisfy our work requirement, we plotted a scatter plot using the matplotlib function available in Python and made a speed noise v/s position noise graph to demonstrate how benign and malicious samples deviate across the noise thresholds (Fig. 1).

From Fig. 1, it is thus clear that Benign samples have the least deviation across the Noise Threshold line and that the Malicious samples have a very high deviation in the position and speed noise.

Algorithm-Filtering Benign Samples:

Input: df (Dataset), noise_threshold, speed_change_threshold, acceleration_change_threshold

Output: benign_samples (Filtered dataset)

1. Initialize an empty list benign_samples.
2. Iterate through each row in df.
3. Check Noise Levels:
  • Ensure |pos_noise_0|, |spd_noise_0|, and |acl_noise_0| are within noise_threshold.
  • Example: If pos_noise_0 = 0.02, spd_noise_0 = -0.03, and acl_noise_0 = 0.05, and noise_threshold = 0.1, this sample is valid.
4. Verify Speed & Acceleration Consistency:
  • Ensure |spd_0 - spd_1| < speed_change_threshold and |acl_0 - acl_1| < accel_change_threshold.
  • Example: If spd_0 = 30, spd_1 = 30.3 and acl_0 = 1.2, acl_1 = 1.3, and thresholds are speed_change_threshold = 0.5, accel_change_threshold = 0.5, then the sample satisfies this rule.
5. Check Positional Alignment:
  • Ensure |pos_0 - pos_1| < |spd_0 - spd_1|, meaning position changes align with speed variations.
  • Example: If pos_0 = 100, pos_1 = 101, and spd_0 - spd_1 = 1, the sample is benign since the position change corresponds to speed variations.
6. Append rows satisfying all conditions to benign_samples.
7. Return benign_samples as the filtered dataset.

## 5 Results and Analysis

• **Dataset statistics**
• **Final balanced dataset**
• **Model performance**

Balanced datasets improved model accuracy by ∼18 %, highlighting the reduction in bias.

## Limitations

a. *Complex Rule Design:* To determine when the activity would be considered benign, some subjectivity and multiple iterations are required to tune the thresholds.
b. *Scalability:* Because of its high demand for memory, it could not process a large dataset; thus, effective chunking was necessary for it.
c. *Synthetic Data Validation:* Accurately reflecting real-world benign behavior by synthesized data was quite challenging.

## Ethics Statement

This research adheres to the ethical standards and guidelines set forth by the relevant institutional review boards and ethics committees. All data used in this study, including the VeReMi dataset, were obtained and processed in compliance with ethical practices. The dataset contains anonymized vehicle communication logs, ensuring no personal information or private data from individuals is disclosed or used without consent.

No human subjects were involved in the research process, and the study did not include any direct interaction with individuals or personal data collection. The experiments and methodologies applied in this paper ensure the privacy and confidentiality of any data utilized.

Furthermore, the authors declare no conflicts of interest regarding this paper's research, authorship, and publication. All results and conclusions presented are based solely on the scientific analysis of the data and are independent of external influence.

## CRediT authorship contribution statement

**Aparup Roy:** Conceptualization, Data curation, Methodology, Writing – original draft, Visualization, Investigation, Formal analysis. **Debotosh Bhattacharjee:** Supervision, Validation, Writing – review & editing. **Ondrej Krejcar:** Funding acquisition, Formal analysis, Writing – review & editing.

## Data Availability

ZenodoPreprocessed VeReMi Dataset for IoV Intrusion Detection (Original data).
